# Quality of antenatal care and associated factors in public health centers in Addis Ababa, Ethiopia, a cross-sectional study

**DOI:** 10.1371/journal.pone.0269710

**Published:** 2022-06-10

**Authors:** Genet Atlabachew Hailu, Zewdu Shewngizaw Weret, Zerihun Adraro Adasho, Belete Melesegn Eshete

**Affiliations:** 1 Department of Midwifery, Menelik II Medical and Health Science College, Kotebe Metropolitan University, Addis Ababa, Ethiopia; 2 Department of Psychiatry, Menelik II Medical and Health Science College, Kotebe Metropolitan University, Addis Ababa, Ethiopia; 3 Department of Neonatal Nursing, Menelik II Medical and Health Science College, Kotebe Metropolitan University, Addis Ababa, Ethiopia; Flinders University, AUSTRALIA

## Abstract

**Background:**

Potentially, the risk of morbidity and mortality during pregnancy and child birth can be prevented through comprehensive, quality antenatal care services. The high maternal mortality rate in developing countries, including Ethiopia, is related to poor quality of antenatal care services and is still a major public health problem. The aim of this study is to assess the quality of antenatal care and associated factors in public health centers in Addis Ababa, Ethiopia.

**Methods:**

An institution-based cross-sectional study was conducted using a quantitative method from December 10 to January 30, 2020. A total of 616 study participants were selected by a systematic random sampling technique. Data was collected using pre-tested structured interview administered questionnaires. The data was entered into Epi-info version 7.2.1 and analyzed by SPSS version 24. Bivariate and multivariable logistic regressions were performed to identify the presence and strength of the association between the outcome and predictor variables.

**Results:**

Overall, 33% of pregnant women received good-quality antenatal care. Satisfaction with antenatal care service, antenatal care initiation time, maintaining confidentiality, and waiting time become significant predictors of the quality of antenatal care. As a result, a lack of confidential care (AOR = 0.37; 95% CI, (0.40, 0.88)), a long waiting time (AOR = 0.6, 95% CI, (0.48, 0.88)), and no satisfaction with ANC services (AOR = 0.26; 95% CI, (0.109, 0.36)) were identified as factors impeding the quality of antenatal care. While starting ANC later than four months of pregnancy was found to be a positive predictor of the quality of antenatal care (AOR = 1.9, 95% CI: (1.21, 3.12)).

**Conclusion:**

Only one-third of pregnant mothers received good quality antenatal care. Lack of confidential care, long waiting time and no satisfaction with antenatal care services were factors hindering the quality of antenatal care. While initiation of antenatal care after four months of pregnancy was a positive predictor of the quality of antenatal care.

## Introduction

During higher the initiation of the sustainable development goal, preventable pregnancy related maternal diseases and death remained higher. As a result, the world health organization (WHO) envisioned that all pregnant woman received timely-appropriate evidence-based quality antenatal care throughout their pregnancy, so that it can save mothers lives [1].

Maternal death remains among the greatest global public health important issues. The world banks estimates that the global burden of sever maternal morbidity is increasing over time and it is higher in low- and middle-income countries than in high income countries. The increment in maternal death would lead to failure to achieve the broad public health goals of improved women’s health and, contribute to bad pregnancy outcome and poor infant health [2].

Through prevention, early detection, and management of pregnancy complications or pre-existing conditions, high-quality antenatal care (ANC) can reduce maternal and neonatal morbidity and mortality, as well as stillbirths [3]. High-quality ANC can influence women’s health seeking behavior towards choosing skilled care at birth, and help them prepare to be able to access it [4]. ANC coverage is a success story in Africa, where 69 percent of pregnant women have at least one ANC contact. However, in order to realize the full life-saving potential of ANC for women and babies, four visits with essential evidence-based interventions and a package known as focused antenatal care are required. Identification and management of obstetric complications, intermittent preventive treatment for malaria during pregnancy, and identification and management of infections such as human immunodeficiency virus, syphilis, and other sexually transmitted infections are all essential interventions in ANC. It is also an opportunity to promote the use of skilled birth attendants and healthy behaviors such as breastfeeding, early postnatal care, and planning for optimal pregnancy spacing, but many of these opportunities are still being missed [5].

Good prenatal care is essential for the mother’s health and the development of the unborn child. Pregnancy is an important time to teach healthy behaviors and parenting skills. Good ANC connects the woman and her family to the formal health system, increases the likelihood of using a skilled attendant at birth, and contributes to overall health throughout the life cycle. Inadequate care during this period disrupts a critical link in the continuum of care, affecting both women and babies [5].

Poor pregnancy outcomes have been linked to insufficient ANC services, both in terms of coverage and quality. The quality of antenatal care services predicts lower maternal mortality and morbidity [6]. Quality ANC services are characterized by service efficiency, timeliness, effectiveness, equity, accessibility, comprehensiveness, acceptability, appropriateness, continuity, privacy, and confidentiality [6].

Although coverage of antenatal care services is increasing, coverage alone cannot ensure the success of ANC services [7]. Aside from ANC coverage, the delivery of quality ANC services and access to these services will have the greatest impact on pregnant women [8]. According to researchers, the high maternal mortality rate in developing countries, including Ethiopia, is linked to poor quality antenatal care services for pregnant women due to three delays. The first delay is due to the time it takes to recognize a problem and decide to seek care, the second delay is due to inaccessibility to a facility, and the third delay is due to inadequacy in receiving adequate and appropriate care [9].

According to the 2016 Demographic and Health Survey, 62 percent of pregnant mothers in Ethiopia received ANC services from skilled providers, and three out of every ten women had four ANC visits [7]. However, the quality of ANC services remains deplorable [10]. The World Health Organization defines ANC services as routine antenatal care, recording general history, assessing individual needs, advice and guidance on pregnancy and delivery, screening tests, self-care education, nutrition, and identification of conditions harmful to health during pregnancy, as well as first-line management and referral provision [11].

To achieve the sustainable development goal in any country, quality is critical factor. Quality is the extent to which a service meets the plan to deliver that service, providing service at a reasonable cost, and fulfilling the client’s expectation. To effectively prevent pregnancy-related problems, the contents of ANC must be revised and its quality be improved [12–16].

Current WHO ANC guideline recommendations focused on antenatal nutrition, maternal and fetal assessment, preventive measures, interventions for common physiological symptom, as well as health systems interventions to improve ANC utilization and quality of care and also the treatment of malaria, tuberculosis and HIV for women during pregnancy [17].

Various studies evaluated the quality of ANC, and the prevalence ranged from 5% to 98.6 percent in India [17]. According to various studies, the quality of ANC was: 43 percent in Nepal [18], 66 percent in urban Slum Aligarh [19], 98.6 percent in Tamil Nadu, India [20], 50 percent in Selangor, Malaysia [15], 17 percent in Pakistan’s Punjab province [21], 71.4 percent in Mexico [22], and 81 percent and 85 percent in Ghana’s Kassena-Nankana and Builsa district health centers [23], and 29 percent in Nigeria [24]. In Ethiopia, 31.5 percent of mothers received good quality ANC in Hossaina town [25], 24.5 percent in North Ethiopia [26], 89 percent in Ambo [27], 48.3 percent in Jimma, South West Ethiopia [28], 52.3 percent in Bahir Dar [29], 41.2% in Sidama region, Ethiopia, and 52.6 percent in Gamo Gofa Zone, southern Ethiopia [30, 31].

The current recommendation for pregnant woman is to attend a minimum of four ANC visit. But, in most developing nations the recommended minimum number of visit is not attended due to poor access and availability of services, communication barrier, shortage of resources and drugs, and limited number of qualified professionals. Mothers’ living area, educational status, parity, gravidity, and time of ANC visit were all factors associated with ANC quality [32–34].

Bringing quality of service requires, intervention done based on evidence generated by rigorous scientific investigation. In Ethiopia, ANC quality studies focused on the number of visits or components of focused antenatal care. Despite the fact that some studies on the quality of ANC have been conducted in various parts of Ethiopia, no published study has been conducted specifically in the capital city (Addis Ababa). As a result, the purpose of this study was to evaluate the quality of antenatal care and identify associated factors in public health centers in Addis Ababa, Ethiopia. As a result, it would fill an existing evidence gap and could serve as a foundation for intervention to improve the quality of antenatal care.

## Materials and methods

### Study setting, population and sample

An institutional-based cross-sectional quantitative descriptive study was conducted at public health centers in Addis Ababa from December 10, 2019 to January 30, 2020. Addis Ababa is Ethiopia’s capital, with ten sub cities and 100 public health centers. According to Ethiopia’s central statistical agency’s population projections for 2014–2017, Addis Ababa city has a total population of 3,434,000 people. There were 1,624,999 men and 1,809,000 women among them. According to Addis Ababa’s health bureau’s 2018 annual report, 454,000 pregnant women received care at public health centers.

The source populations were all pregnant women attending antenatal care services at Addis Ababa’s public health centers. The study included pregnant women who visited specific public health centers during the data collection period, while critically ill ANC attendants were excluded.

A sample size of 616 was calculated using a single population proportion formula, considering the 95 percent confidence interval, the 5 percent marginal error (d), and the prevalence of ANC services quality in Harar, 24.3 percent [14], as well as the design effect of two and a 10% non-response rate. A multistage sampling technique was used. To begin, using simple random sampling techniques, five sub-cities (Aarada, Addis-ketema, Akaki-kality and Nefas-silk lafto sub cities) were chosen from a total of ten. Second, 30 public health centers (Abebe Bikila, Abyssinia, Addis Ketema, Addis Raye, Felege-meles, Ginbot 20, woreda 01, woreda 02, woreda 05, woreda 011, woreda12, woreda 03, Gelan, Kality, Klinto, Saris, Selam-frea, Serti, Adisu-Gebeya, Selam, Maychew, Shromeda, Entoto-Fana, Hidasse, Arada, Churchill, Janmeda, Kebena, Semen and Ras-emeru health centers) were selected from each selected sub-city using simple random sampling technique. The sample size was distributed to the selected health centers using a proportional allocation based on population size. After the first pregnant woman was chosen by lottery method, systematic random sampling technique was used to select study participants from each health center.

### Eligibility criteria

All pregnant women attending antenatal care services in the selected health centers during data collection period were included in the study, while critically ill pregnant women who can’t respond were excluded from the study.

### Study variables

#### Dependent variable

Quality of antenatal care.

#### Independent variables

*Socio-demographic characteristics*. Maternal age, educational status, and monthly income.

*Process*. Interpersonal communication, technical skills of professionals, confidentiality and respectful care, antenatal visits and waiting time during ANC visits, provision of adequate information, and appointment scheduling.

*Structure*. Physical setting of the health facility, availability of ANC guideline, waiting area, resting room, visual privacy, separate ANC examination room, hand washing facility and client satisfaction.

### Operational definition

#### Good quality of ANC services

If a health care facility provides 75 percent of the required focus antenatal care components of services, such as physical examination, basic diagnostic laboratory service, therapeutic drugs, information on danger signs, birth preparation, and advice [13].

#### Poor-quality of ANC services

If the health institution provides less than 74% of the necessary focus antenatal care components of services, they do not provide physical examinations, basic diagnostic laboratory services, therapeutic drugs, information provided on danger signs, birth preparedness, and advice [13].

### Data collection tools

Data was collected using a structured interview administered questionnaire developed after reviewing various literature sources [12, 14]. Furthermore, for additional information, the client’s medical records were reviewed to double check the data collected through interview. The questionnaires were written in English, translated into the local language (Amharic), and then back to English for consistency. It has four parts, part one is about socio-demographic and obstetrics information, part two is about process related information, part three is about structural aspect of services, part four about performance observational checklist. To ensure the quality of the data, the questionnaire was arranged sequentially using simple, clear, short, and acceptable language, and it was pre-tested on 5% of mothers attending ANC services at non-study health centers. Some changes were made to the unclear and difficult questions identified during the pre-test. To determine tool validity, content validity is used. Cronbach’s alpha was computed, and the coefficient scale was 0.89.

### Data collection procedures

Six nurses with diploma level qualifications collected the data, while three bachelor degree holder nurses supervised the process. Data collectors arrived early in the morning during the data collection period to give clients reception cards to record their time of arrival at the health center. Data collectors and supervisors received one day of training. On-site assistance was provided by the investigators. The principal investigator categorized and coded the collected data. The questionnaires were reviewed and checked for completeness, consistency, and legibility by the supervisor and principal investigator during data collection, and feedback was provided to data collectors every morning.

### Data processing and analysis

The collected data was checked for completeness and consistency. Then it was coded, entered, cleaned, and stored in Epi-info version7.2.1 and exported into SPSS version 24 for analysis. Descriptive statistics including frequencies, means, medians, and standard deviations were calculated. The results were presented in the form of tables, graphs and text.

To assess the association of variables, bivariate analysis was carried out. Each variable was first analyzed by using bivariate logistic regression, and independent variables having a P-value < 0.2 were entered into the multivariable logistic regression model. Multivariable logistic regressions were carried out to identify the most important predictor variables with quality of ANC service and controlling the effect of confounding variables. Finally, variables with a p-value <0.05 were considered statistically significant.

### Ethical clearance

The study was carried out after the Kotebe Metropolitan University Menelik II medical and health Sciences College’s institutional ethical review board (IERB) committee reviewed and approved it with (IERB number-አጠ5/32/10/4812). Data collectors were instructed on how to handle sensitive and emotional issues, as well as the importance of confidentiality. Prior to data collection, a letter of permission was given to and accepted by the Addis Ababa Health Bureau, the purpose and procedure for data collection were clarified, and confidentiality and privacy were guaranteed. All participants provided verbal informed consent and were informed that their participation was entirely voluntary.

## Results

### Socio-demographic characteristics

The study included 609 samples out of a total of 616, for a response rate of 98.8%.

Concerning age, 41.9% of respondents were between the age groups 26–31 years old. Among the participants, 224 (36.4%) completed secondary school, and 15.4% of them were unable to read and write. More than half (62.2%) of participants earn up to 1000 Ethiopian birr per month (Table 1).

**Table 1:** Socio-demographic characteristics of women attending ANC services in public health centers in Addis Ababa, Ethiopia, 2020 (n = 609).

Variables	Category	Frequency	Percent
**Age in year**	≤19	23	4
	20–25	201	33
	26–31	255	41.9
	32–37	106	17.4
	≥38	24	4
**Educational status**	Illiterate	94	15.4
	Primary	163	26.8
	Secondary	224	36.4
	Vocational and above	128	21
**Marital status**	Married	565	92.8
	Unmarried	44	7.2
**Occupation**	Employed	192	31.5
	Unemployed	39	6.4
	Housewife	378	62.1
**Religion**	Orthodox	283	46.5
	Muslim	246	40.4
	Protestant	80	13.1
**Monthly income in Ethiopian Birr**	≤1000	379	62.2
	1001–2000	71	11.7
	2001–3000	46	7.6
	≥3001	113	18.6

### Quality of antenatal care

**Fig 1:**
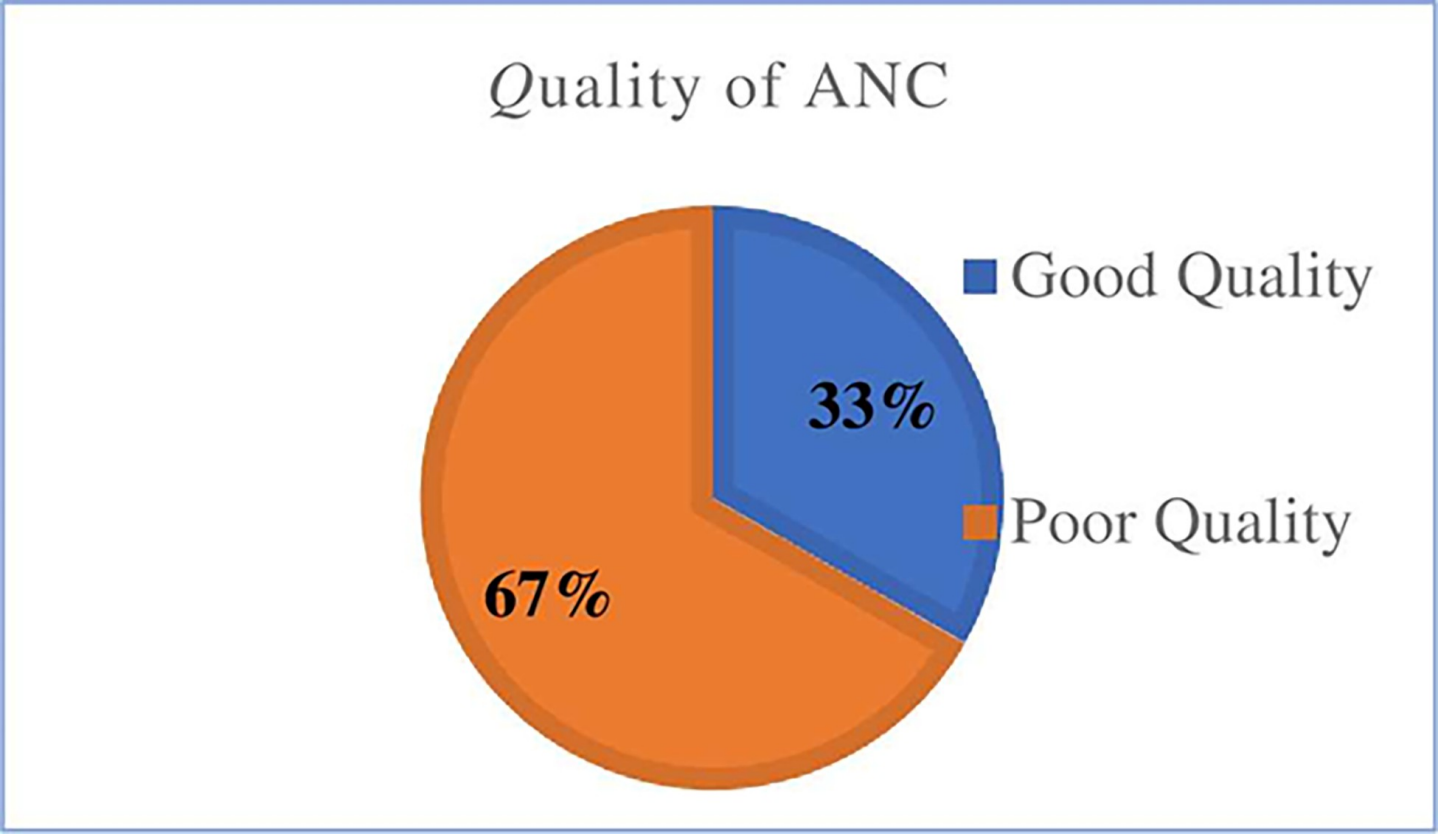
Quality of ANC service in selected public health centers in Addis Ababa, Ethiopia, 2020 (n = 609).

In terms of antenatal care service quality, 203 (33%) of pregnant women received good quality antenatal care, while the remaining 406 (67%) received poor quality antenatal care (Fig 1).

### Process and satisfaction related factors

Concerning satisfaction, 433 (71.1) of mothers were satisfied with the ANC service they received, and 176 (28.9) were not satisfied. Among total participants, 317 (52%) started ANC follow-up at four months of gestational age, whereas 216 (35.5%) started antenatal care services visits before or at three months of gestational age. Among the total participants, 138 (22.7) had four ANC visits. A majority (74.2%) of the women were effectively communicating with healthcare providers, and confidentiality was maintained for 380 (62.4%) of the women (Table 2).

**Table 2:** Process and satisfaction related to ANC service provided for pregnant women attending in public health centers in Addis Ababa, February, 2020 (n = 609).

Variables	Category	Frequency	Percent (%)
Satisfaction with ANC service	Satisfied	433	71.1
	Unsatisfied	176	28.9
Providing respectful care	Yes	382	62.7
	No	227	37.3
Number of ANC visit	One	95	15.6
	Two	181	29.7
	Three	195	32
	≥ Four	138	22.7
ANC starting time in months	≤ 3 months	216	35.5
	4 months	317	52
	≥ 5 months	76	12.5
Providing confidential care	Yes	380	62.44
	No	228	34.4
Interpersonal communication skill	Effective	452	74.2
	Non-effective	156	25.6
Waiting time	<60minutes	187	30.7
	≥60 minutes	422	69.3
Appointment	Yes	588	96.6
	No	21	3.4
Advise on complication during pregnancy	Yes	430	70.6
	No	179	29.4
Advise on birth plan	Yes	183	30
	No	426	70
Professional’s skill	competent	285	46.8
	Not-competent	324	53.2

On bivariate logistic regression analysis, six variables, namely: satisfaction with ANC service, providing respectful care, ANC start time, maintaining confidentiality, interpersonal communication skills, and waiting time, had a statistically significant association with the quality of antenatal care at p-value < 0.2. However, on multivariate logistic regression analysis, only four variables, namely satisfaction with ANC service, ANC initiation time, maintaining confidentiality, and waiting time, became significant predictors of the quality of antenatal care. As a result, lack of confidential care (AOR = 0.37; 95% (0.40, 0.88)), long waiting time (AOR = 0.6, 95% CI, (0.4,0.88)) and no satisfaction with ANC services (AOR = 0.26; 95% CI, (0.109, 0.36)) were found factors hindering the quality of antenatal care. While initiation of ANC after four months of pregnancy was found to be positive predictor of quality of antenatal care (AOR = 1.9, 95% CI: (1.21, 3.12)) (Table 3).

**Table 3:** Crude and adjusted odds ratios from logistic regression analysis of factors associated with quality of ANC service among pregnant women attending public health centers in Addis Ababa, February, 2020 (n = 609).

Variable	Category	Quality of ANC		COR (95% CI)	AOR (95% CI)
		Poor (%)	Good (%)		
Satisfaction with ANC Service	Satisfied	247(40.6)	186(30.5)	1	1
	Unsatisfied	159(26.1)	17(2.8)	*0*.*14(0*.*08*,*0*.*24)*	*0*.*26(0*.*109*,*0*.36) *
Respectful care	Yes	144(23.6)	238(39.1)	1	1
	No	59(9.7)	168(27.6)	*1*.*7(1*.*2*,*2*.*5)*	1.3(0.89,1.97)
ANC imitation time	≤3 months	58(9.5)	158(25.9)	1	1
	4 months	128(21.0)	189(31.0)	*1*.*8(1*.*27*,*2*.*68)*	*1*.*9(1*.*21*,*3*.12) *
	≥5 months	17(2.8)	59(9.7)	0.78(0.42,1.46)	0.98(0.44,2.17)
Maintaining confidentiality	Yes	241(36.6)	140(23.)	1	1
	No	165(27.1)	65(10.3)	*1*.*5(1*.*6*,*2*.*17)*	*0*.*37(0*.*17*,*0*.81*
Interpersonal communication	Effective	275(45.2)	177(29)	1	
	Non-Effective	130(21.3)	26(4.3)	*3*.*2(2*.*03*,*5*.*105)*	1.67(0.99,2.8)
Waiting time	< 1hour	103(16.9)	84(13.8)	1	1
	≥ 1hour	303(49.8)	119(19.5)	*0*.*48(0*.*34*,*0*.*69)*	*0*.*6(0*.*4*,*0*.88) *

COR, Crude Odds Ratio; AOR, Adjusted Odds Ratio

*—Variables which had statically significant association on multivariate logistic regression.

## Discussion

According to the findings of this study, 33.3 percent of mothers received high-quality ANC services. It was consistent with the findings of study in Hossaina Town, Ethiopia 31.5% [25]. It was, however, higher than the one in studies conducted in Pakistan’s Punjab province 17% [21], and North Ethiopia 24.5% [26]. And it was much higher when compared to studies conducted in Nigeria, where it was 5% [24]. The disparity could be due to a difference in the tools used, difference in the customer’s perception and expectations of quality and/or difference in the socioeconomic status of the participants. It could also be due to a difference in the cutoffs used to differentiate between poor and good quality antenatal care.

It was found to be lower than studies conducted in Nepal 43% [18], urban Slum Aligarh 66% [19], Tamil Nadu, India 98.6% [20], Selangor, Malaysia 50% [15], Mexico 71.4% [22], 81% and 85% in Ghana’s Kassena-Nankana and Builsa district health centers [23], Ambo, Ethiopia 89% [27], Jimma, South West Ethiopia 48.3% [28], Bahir Dar, Ethiopia 52.3% [29], 41.2% Sidama region, Ethiopia [30] and Gamo-Gofa, southern Ethiopia 52.6% [31]. The inconsistency could be attributed to a difference caseloads/ number of ANC service receiving because if there is high patient flow, the waiting time would be longer and it might have been affected the number clients receiving quality ANC service.

Concerning factors associated with quality of antenatal care, four variables, namely satisfaction with ANC service, ANC initiation time, maintaining confidentiality, and waiting time, become significant predictors of quality of antenatal care. The likelihood of receiving good-quality ANC was reduced by 63% among pregnant women who did not maintain confidentiality when compared to women who did (AOR = 0.37, 95% (0.40, 0.88)). Pregnant women who waited more than one hour were 40% less likely to receive quality ANC than women who waited less than one hour (AOR = 0.6, 95% CI, (0.40, 0.88)). This might be explained by clients having problems reaching a health institution and not receiving service soon, which in turn might affect clients’ perception of quality care. Women who started ANC visit after four months of gestation were two times more likely to receive good quality ANC service when compared to women who started their first ANC visit at 3 months or earlier gestational age (AOR = 1.9; 95% CI, (1.21, 3.12)).

WHO recommends that pregnant women should start their first ANC visit before 4 months, and in this study, 35.5% of pregnant mothers started antenatal care in the first three months, which is higher compared to the other studies conducted in the Gamo-Gofa Zone, which is 22.5%, and lower than that of the study carried out in Bahir-Dar, which is 37.9% [6, 29]. This might be due to problems in access to the health facilities. This study found that 94.5% of mothers received iron supplementation, 90% of pregnant mothers received TT immunization, and 97.7% of pregnant mothers received urine tests, which was higher than study conducted in Gondar; 35% urine test, 45% TT immunization, and 64% iron supplementation, and urine tests done at 15.6% in Kenya [8, 26]. This inconsistency might be due to the fact that the current study was conducted in the capital city, Addis Ababa. The health facilities might be equipped with the necessary tests and drugs supplied to pregnant mothers.

### Strength and limitations of the study

#### Strength of the study

A large sample size was used for the analysis and the study used interview and medical records review to double check the data collected.

#### Limitation of the study

The study used only quantitative methods, if it was triangulated with qualitative data strong evidence can be generated.

## Conclusion

In this study, only three out of every ten pregnant mothers received high-quality antenatal care, indicating that mothers who received quality antennal care are low, and more effort is required. This research showed a variety of factors that contributed to poor antenatal care quality, including a lack of confidentiality during care, long waiting times, and unsatisfactory antenatal care. The management of health centers and regional health offices should improve the process components of providing ANC services like minimizing the waiting time and modifying service rooms in a way that can maintain client’s confidentiality and privacy. While providing services the care givers should maintain confidentiality and reduce the waiting time of clients. Antenatal care programs aimed at increasing mothers’ satisfaction should be designed. Further studies should be conducted on the barriers and facilitators of providing quality antenatal care.

## Supporting information

S1 File Survey questionnaire. (PDF)

S2 File Data availability. (SAV)

## References

[1] WHO Recommendations on Antenatal Care for a Positive Pregnancy Experience. Geneva: World Health Organization; 2016. . 28079998

[2] Geller S.E., Koch A.R., Garland C.E. et al. A global view of severe maternal morbidity: moving beyond maternal mortality. Reprod Health 15, 98 (2018). 10.1186/s12978-018-0527-2 PMC6019990 29945657

[3] WHO statement on antenatal care, http://www.who.int/maternal child adolescent /documents/rhr_11_12/en/

[4] Chukwuma A, Wosu AC, Mbachu C, Weze K. Quality of antenatal care predicts retention in skilled birth attendance: a multilevel analysis of 28 African countries. BMC Pregnancy Childbirth. 2017; 17:152 doi: 10.1186/s12884-017-1337-1 28545422 PMC5445515

[5] Lincetto O, Mothebesoane-Anoh S, Gomez P, Munjanja S. Chapter 2; Antenatal Care: Opportunities for Africa’s Newborns. Geneva, Switzerland: WHO; accessed from http://www.who.int

[6] Makonnen N, Berheto TM, Ololo S, Tafese F. Quality of antenatal Care Services in Demba Gofa woreda, Med Pub Journals. 2017;1–18

[7] Agency CS. Federal Democratic Republic of Ethiopia Demographic and Health Survey. 2016. 1–59.

[8] Van Eijk AM, et al. Use of antenatal services and delivery care among women in rural western Kenya: A community based survey. Reprod Health. 2006;3:1–9. 16597344 10.1186/1742-4755-3-2 PMC1459114

[9] Legesse T. Trends and causes of maternal mortality in Jimma University Specialized Hospital.Int women heath. 2017;307–13. doi: 10.2147/IJWH.S123455 28496370 PMC5422567

[10] Gebremariam A. Causes of maternal death in Ethiopia between 1990 and 2016: systematic review with meta-analysis. 2019;.

[11] Tekelehaymanot G. Assessment of quality of antenatal care Service provision and associated factor at governmental health facilities of Harar town. 2018;6(4).

[12] Akachi Y. and Kruk M. E., “Quality of care: measuring a neglected driver of improved health,” Bulletin of the World Health Organization: Policy & Practice, 2017(96) (6)465–472. 10.2471/BLT.16.180190 PMC5463815 28603313

[13] Mainz J., “Defining and classifying clinical indicators for quality improvement,” International Journal for Quality in Health Care, 2003(15)(6) 523–530. doi: 10.1093/intqhc/mzg081 14660535

[14] Nylenna M., Bjertnaes O., Saunes I. S., and Lindahl A. K., “What is good quality of health care?” Professions and Professionalism, 2015(5)(1)1–15.

[15] Yeoh P. L., Hornetz K., and Dahlui M., “Antenatal care utilization and content between low-risk and high-risk pregnant women, PLoS ONE, 2016 (11)(3). 10.1371/journal.pone.0152167 PMC4807004 27010482

[16] Beeckman K., Louckx F., Masuy-Stroobant G., Downe S., and Putman K., The development and application of a new tool to assess the adequacy of the content and timing of antenatal care,” BioMed Central Health Services Research, 2011(11) (213) 1–10. doi: 10.1186/1472-6963-11-213 21896201 PMC3176177

[17] Barreix M, Lawrie T, Kidula N, Tall F, Bucagu M, Chahar R, et al. Development of the WHO Antenatal Care Recommendations Adaptation Toolkit: A standardized approach for countries. Health Research Policy and Systems. 2020; 18. doi: 10.1186/s12961-020-00554-4 32564777 PMC7310220

[18] Bastola P., Yadav D. K., and Gautam H., “Quality of antenatal care services in selected health facilities of Kaski district, Nepal,” International Journal of Community Medicine and Public Health, 2018(5)(6) 2182–2189.

[19] Mehnaz S., Abedi S., Fazli Z., Ansari M., Mohammed, and A. Ansari, “Quality of care: predictor for utilization of ANC services in slums of Aligarh,” International Journal of Medical Science and Public Health, 2016 (5) (9)1869–1873.

[20] S. G., “Quality of antenatal care services at sub-centers: an infrastructure, process and outcome evaluation in a district in Tamil Nadu,” International Journal of Community Medicine and Public Health, 2017 (4) (11)4071–4077.

[21] Majrooh M. A., Hasnain S., Akram J., Siddiqui A., and Memon Z. A., Coverage and quality of antenatal care provided at primary health care facilities in the “Punjab” province of Pakistan PLoS One, 2014 (9) (11). 10.1371/journal.pone.0113390 PMC4237449 25409502

[22] Heredia-Pi I., Servan-Mori E., Darney G., Reyes-Morales H., and Lozano R., “Measuring the adequacy of antenatal health care: a national cross-sectional study in Mexico,” Bulletin of the World Health Organization, 2016 (94) (6) 452–461. 27274597 10.2471/BLT.15.168302 PMC4890208

[23] Duysburgh E., Williams A., Williams J., Loukanova S., and Temmerman M., “Quality of antenatal and childbirth care in northern Ghana, An International Journal of Obstetrics & Gynecology, 2014 (121) (54) 117–126. 10.1111/1471-0528.12905 25236645

[24] Fagbamigbe A. F. and Idemudia E. S., “Assessment of quality of antenatal care services in Nigeria: evidence from a population-based survey, Reproductive Health, 2015(12)(88) 1–9. doi: 10.1186/s12978-015-0081-0 26382228 PMC4574449

[25] Muchie K. F., “Quality of antenatal care services and completion of four or more antenatal care visits in Ethiopia: a finding based on a demographic and health survey,” BMC Pregnancy Childbirth, 2017(17) (300). doi: 10.1186/s12884-017-1488-0 28893222 PMC5594613

[26] Fesseha G., Alemayehu M., Etana B., Haileslassie K., and Zemene A., “Perceived quality of antenatal care service by pregnant women in public and private health facilities in Northern Ethiopia,” American Journal of Health Research, 2014(2) (4) 146–151.

[27] Yabo A. N., Gebremicheal M. A., and Chaka E. E., “Assessment of quality of antenatal care service provision among pregnant women in Ambo town public health institution, Ambo, Ethiopia, 2013,” American Journal of Nursing Science, 2015(4)(3) 57–62.

[28] Abate T. M., Salgedo B. S., and Bayou N. B., “Evaluation of the quality of antenatal care (ANC) service at higher 2 health center in Jimma, South west Ethiopia,” Open Access Library Journal, 2015(2)1398:1–1398:9.

[29] Ejigu T., Woldie M., and Kifle Y., “Quality of antenatal care services at public health facilities of Bahir-Dar special zone, Advances in Public Health Northwest Ethiopia,” BMC Health Services Research, 2013(13) (443)1–443:8. 24161007 10.1186/1472-6963-13-443 PMC4231347

[30] Kare AP, Gujo AB, Yote NY. Quality of antenatal care and associated factors among pregnant women attending government hospitals in Sidama Region, Southern Ethiopia. SAGE Open Medicine. January 2021. doi: 10.1177/20503121211058055 34868590 PMC8640313

[31] Makonnen N., Berheto T. M., Ololo S., and Tafese F., “Under nutrition and its association with infant and young child feeding summary index among 6 to 23 Months in demba Gofa Woreda, southern Ethiopia,” Journal of Nutritional Health & Food Science, 2017(5) (3); 1–15.

[32] Islam M. M. and M. S. & Masud, “Determinants of frequency and contents of antenatal care visits in Bangladesh: assessing the extent of compliance with the WHO recommendations,” PLoS One, 2018 (13) (9), Article ID e0204752. 10.1371/journal.pone.0204752 PMC6160162 30261046

[33] Dansereau E., McNellan C. R., Gagnier M. C. et al., “Coverage and timing of antenatal care among poor women in 6 Meso-american countries,” BMC Pregnancy Childbirth, 2016 (16) (234). 10.1186/s12884-016-1018-5 PMC4991111 27542909

[34] Tadesse Trhas, Maud Lebitsi, Assessment of Quality of Antenatal Care Services and Its Determinant Factors in Public Health Facilities of Hossaina Town, Hadiya Zone, Southern Ethiopia: a longitudinal study, Advances in Public Health 2020(2020)11, 10.1155/2020/5436324

