# Supplementary material for: Quality of antenatal care and associated factors in public health centers in Addis Ababa, Ethiopia, a cross-sectional study
Source: PLoS One. 2022 Jun 10;17(6):e0269710. doi: 10.1371/journal.pone.0269710 (PMC9187099; doi:10.1371/journal.pone.0269710)
Supplement: S1 File — (PDF) [file pone.0269710.s001.pdf]

Questionnaire

INFORMATION SHEET

Kotebe Metropolitan University Menelik II medical and health Sciences College in track of health service management.

How are you? -- I am here to collect data for a project titled Quality of Antennal Care Services and Associated factors among pregnant women attending public health centers, Addis Ababa, Ethiopia. As recommended by the researcher/data collector and to answer that question. For the accomplishment of this study, you are kindly requested to respond genuinely and voluntary with patience. This will help us to improve quality antenatal care services at health facilities based on your answers to our questions. Your name will not be written in this form. All information given by you will be kept strictly confidential. Your participation is voluntary and you are not obligate to answer any question you do not wish to answer. If you fill discomfort with the interview, please fill free to drop it any time you want. Thank you very much for your willingness to listen to me. In case, if you have any question you can ask:

Contact address: -Genet Atlabachew, cell phone: 09-11-95-19-69.

Name of data collector________________________ Signature__________ date________

Thank you!!

Exit- interview questioner

Write Tick or number, or statements or word of the interviewed mother in front of the question of space provided ___ of this space

part one: - Socio-demographic and obstetrics information

101. Age of Client ________ yrs.

102 What is your religion? 1. Orthodox 2. Muslim 3. Protestant 4. Catholic

103. Marital Status 1. Married 2. Unmarried 3. Divorced 4. Widowed 5. separated

104. Educational Status 1. No formal education 2. Primary school 3. Secondary school

4. Diploma 5. Degree 6. Masters

105. What is your Occupational? 1. Government employee2. Non-governmental employee 3. Private employee 4. Students 5. Daily laborer 6. House wife

106. Income level per month: ____________________Ethiopian birr

107. Number of Pregnancy: __________________

108. Is the pregnancy planned? 1. Yes 2. No

109. How many visits do you have in this pregnancy?

1. First visit 2. Second visit 3. Third visit 4. More third visit

1010. Duration of pregnancy1. ____month 2. _weeks 3. Don't know

1011. When was your first visit? 1.---- Date/2. ------ Months 3. don't know

1012. Why did you start visit at this time?

1. TT immunizations 2. Antenatal checkup 3. Pregnancy test 4. Other medical cases

Part two: process related information

1013. Does the health care provider perform general examination in this visit?

1. Yes 2. No

1014.Q-1013 if your answer yes which one?

1. Weight measured 1. Yes 2. No 3. I Don't know

2. Pallor evaluated 1. Yes 2. No

3. BP measurement 1. Yes 2. No 3.I Don't know

4. Edema evaluated 1. Yes 2. No 3. I Don't know

5. Ultrasound? 1.Yes 2.NO 3. I Don't know

1015. Dose the health care provider explains about the examination?

1. Yes 2. No 3. I cannot remember

1016.Q1015 If yes, did the health care provider explain about the result of examination before?

1. Yes 2. NO 3. I cannot remember

1017. Below the following the health provider performs Laboratory investigation

1. Hemoglobin/Hematocrit 1. Yes 2. No

2. VDRL 1. Yes 2. No

3. Blood group 1. Yes 2. No

4. RH factor 1. Yes 2. No

5. Urine test 1. Yes 2. No

6. HIV test 1. Yes 2. No

7. Stool examination 1. Yes 2. No

1018. Did you take the iron tablets? 1. Yes 2. No

1019. If QNO 1018yes Why (for what benefits) did you take the iron tablets?

1.To prevent anemia 2. Make you stronger

3.No need to take 4. Don't know

1020.During this pregnancy, did you take Tetanus toxoid immunization?

1. Yes 2. No 3. Don't know

1021. If yes for Q#1020, how many times did you get a tetanus injection? ---

1022. Was the discussion b/n the health care provider and you was confidential.

1. Yes 2. No 3. I am not sure

1023. Did the health care provider treat you respectfully?

1. Yes 2. NO 3. Don't know

1024. After you arrived in the health facility how much time did you spent to get the health care provider.

1. Minutes ____2. Hour's __3. Day---- 4. I cannot remember

1025. What did you say about the time that you spent?

1. Very dissatisfied 2. Dissatisfied 3. Neutral 4. Satisfied 5. Very satisfied

1026. Is there any time that you return to your home without having checkup?

1. Yes 2. No 3. I cannot remember

1027. If yes for Q#1026 explain the reason

1. Lack of health care provider 2. Lack of laboratory Equipment 3. Due to taking long time 4. Others(specify)__________

1028. During your follow up most of your visits were done by how many health professionals 1-one health professional 2-Two-professional 3-More than two

1029. How much you are satisfied with the above care.

1.Very dissatisfied 2. Dissatisfied 3. Neutral 4. Satisfied 5. Very Satisfied

1030. Which group of health professional is mostly giving you ANC

1-Midwife 2-Nurse 3-Doctor4.Health officer 5-Both Nurse &midwife 6. I don't know

1031. From the below-mentioned health care professional whom groups did you preferred

1-Mid wife 2-Nurse 3-Doctor4.Health officer 5-Both Nurse &mid wife 6. I don't know

1032. During your follow up do the health professional explain about the following points in Satisfactory way

1.-Type of laboratory investigation 1. Yes 2. No3. I don't know

2 -Complication that occurs during pregnancy 1. Yes 2. No3. I don't know

3-Bleeding 1. Yes 2. No 3. I don't know

4-Head ach & blurred vision 1. Yes 2. No3. I don't know

5 -True sign of labor 1. Yes 2. No3. I don't know

6-When to go to health facilities for delivery 1. Yes 2. No3. I don't know

7. Pre-preparation before delivery 1. Yes 2. No3 I don't know

1033. About your antenatal follows up on which points do you agree.

1033-1. We were not waiting for Long time to get health workers.

1. Strongly disagree 2. Disagree 3. Neutral 4. Agree5.You are strongly agreeing

1033-2. The follow up would be good conducted by one health professional.

1. Strongly disagree 2. Disagree 3. Neutral 4. Agree5. strongly agree

1033-3. Health care providers were usually initiating to ask question.

1. Strongly disagree 2. Disagree3. Neutral 4. Agree5. strongly agree

1033-4. They give as enough time to ask question.

1. Strongly disagree 2. Disagree3. Neutral 4. Agree 5. strongly agree

1034. During (any of) your Antenatal care visit(s) where you told about the signs of pregnancy complications or danger sign of pregnancy?

1. Yes 2. NO 3. I cannot remember

1035. If Q. No1034 yes, which one of the following the topics.

1.Vaginal bleeding 1. Yes 2. NO 3. I cannot remember

2, Vaginal gush of fluid. 1.Yes 2. No 3. I cannot remember

3.Severe headache 1. Yes 2. No 3. I cannot remember

4.Blurred vision. 1.Yes 2. No 3. I cannot remember

5.Fever 1. Yes 2. No 3. I cannot remember

6.Abdominal pain. 1.Yes 2. No 3. I cannot remember

7.Convulsion 1. Yes 2. No 3. I cannot remember

1036.During any of your antenatal visit where you told about birth preparedness plan?

1.Yes 2. No 3. Don't know

1037.If Q.NO1036 Yes, which plans where you told about?

1.Place of birth 1. Yes 2. No 3. Don't know

2.Supplies needed for birth 1. Yes 2. No 3. Don't know

3.Emergency transportation 1. Yes 2. No 3. Don't know

4.Money/emergency fund 1. Yes 2. No 3. Don't know

5.People to support during/after birth 1. Yes 2. No 3. Don't know

6.Potential blood donors 1. Yes 2. No 3. Don't know

1038. Generally how much did you satisfied with advice you had in this visit?

1. Very dissatisfied 2. Dissatisfied 3. Neutral 4. Satisfied 5. Very satisfied

1039. How much did you pay for the care that you receive _________Birr?

1040. How much expensive the money that you pay

1-Very expensive 2-Appropriate 3-Minimume4.None

1041. Where did you want to give birth

1-In this health center 2-Other health center 3-Home 4-Hospital

1042. Q1041 if your choice is number one (1) what are the reason among the following?

Q1041 if your choice is number one the reason?

1- It is near to my house

2-I like the health care provider

3-Health care providers provide good care

4-Better medical equipment are available

5-I usually give birth in this specific place

6. The waiting time was very short

1043. Is the health care provider gives you appointment. 1. Yes 2. No

1044. To have a good ANC which of the followings needs to be improved

1- Increase health care provider 2- Drug supply 3-Laboratory

4-Cleanness of the room 5-Short waiting time 6-Extra rooms

1045. Today or previously, did you receive a health advice? 1-Yes 2- No

1046. Q1045 if-yes tell the topic that you receive

1-HIVS/AIDS 1. Yes 2. NO 3. Don't know

2-STDS 1. Yes 2. No 3. Don't know

3-nutrition 1.yes 2. No 3. Don't know

4-ANC 1. Yes 2. No 3. Don't know

5-Family planning 1. Yes 2. No 3. Don't know

6-Vaccination 1. Yes 2. No 3. Don't know

1047. How much did you get satisfied with the advice given?

1. Very dissatisfied 2. Dissatisfied 3. Neutral 4. Satisfied 5. Very satisfied

1048. Among the care that you receive which one is very good

1. Counseling service

2. TT Vaccinations

3. HIV testing and counseling

4. Laboratory activities

1049. During your follow up is there any obstacle? 1. Yes 2. No

1050. How much did you satisfy about the follow up you had up to this time.

1. Very dissatisfied 2. Dissatisfied 3. Neutral 4. Satisfied 5. Very satisfied

1051. Did you receive the care that you want and need? 1. Yes 2. No

Part III: Structural aspect of services

Health unit

**1**	Does ANC unit have plan document	1.YES<br>2.NO
2	Does the health institution have prepared action plan for the current budget for ANC.	1.Yes<br>2. No
3	There clear job description for ANC unit staff in this health institution	1.yes<br>2. No

Logistics

1	Does this health institution have transportation facility?	1.Ambulance<br>2. motorcycle<br>3. Car<br>4. none
2	If above are available, are they functional?	1.Yes 2.NO
3	Does the health institution have stand by generator? If no What alternative means do you use _______________	-----
4	Does the health institution has enough budget for vehicle maintenance and fuel	1.Yes 2.NO
5	What water source is using	
	Pip water	1.Yes 2.NO
	Well Water	1.Yes 2.NO
**6**	**Waiting space**	
	Protected waiting area	1.Yes 2.NO
	Adequate sitting space	1.Yes 2.NO
**7**	**ANC / Consultation room**	
	Separate room	1.Yes 2.NO
	Auditory privacy	1.Yes 2.NO
	Visual privacy	1.Yes 2.NO
	Registration book and referral book	1.Yes 2.NO

	Clinical management guide line	1.Yes 2.NO
	Examination Table/couch	1.Yes 2.NO
	Fetal stethoscope (Pinard)	1.Yes 2.NO
	Measuring Tap	1.Yes 2.NO
	Weighting scale	1.Yes 2.NO
	Stethoscope & Blood pressure Apparatus	1.Yes 2.NO
	Maternal & child health cards (FANC).	1.Yes 2.NO
	Thermometer	1.Yes 2.NO
**8**	**Facilities sanitation**	
	Toilet	1.Yes 2.NO
	Hand wash room with Soap	1.Yes 2.NO
	Electricity	1.Yes 2.NO
	Drinking water facility	1.Yes 2.NO
**9**	**Laboratory Facilities and medical Supplies**	
	Syphilis test	1.Yes 2.NO
	Urine Analysis test	1.Yes 2.NO
	HIV test	1.Yes 2.NO
	HBSAg	1.Yes 2.NO
	Pregnancy test	1.Yes 2.NO
	Hemoglobin test	1.Yes 2.NO
	Albumin	1.Yes 2.NO
	CBC (WBC. differential)	1.Yes 2.NO
	IFA tablets are available	1.Yes 2.NO
	TT Vaccines available	1.Yes 2.NO

Sterilization

1	Sterilization is done by: -	1. Dry oven<br>2. Autoclave<br>3. Boiling
2	Infection prevention measure taken	1.yes<br>2.No
3	If no for#2 state the reason	-----------
4	Hand washing facility with soap	1.yes 2. No
5	Decontamination	1.yes 2. No
6	Surgical Gloving	1.yes 2. No
7	Cleaning gloving	1.yes 2. No
8	Heavy duty gloving	1.yes 2. No
9	High level disinfection	1.yes 2. No
10	Alcohol-based glycerin hand rub	1.yes 2. No
11	Puncture-proof container	1.yes 2. No

Part IV: Performance Observational Checklist

s.n	ANC Observation checklist	Yes	No	Remark
1	washing facilities water, soap, and towel are available			
2	Observe greets and calls client by her name and introduce her /himself.			
3	Observe reviews clinic record before starting the session and check about previous pregnancy, number, and outcome.			
4	Observe comprehensive history taking.			
5	Observe take pulse rate, blood pressure, and temperature and Measured weight.			
6	Observe breast Examination: - inspection, Palpation & Preparation for breast feeding.			
7	Observe examine skin, conjunctivae, and legs for edema, redness, and varicose veins, thyroid, mouth, breast and lungs.			
8	Observe palpates uterus and perform maneuvers to detect fetal position and situation and measure uterine height and listens to the fetal heart rate (>18 wks).			
9	Observe determines weeks of gestation, expected date of delivery progress of pregnancy.			
10	Observe informs mothers about her and fetus's health condition.			
11	Observe informs mothers about any complication and management.			
12	Observe orients women for the place of delivery (health centers, hospital).			

13	Observe orients women about personal hygiene, rest, exercise and general care.			
14	Observe orients women about STI and HIV/AIDS prevention.			
15	Observe counseling about harmful habits likes; smocking, drug abuse, alcoholism and traditional herbs to induce labor.			
16	Observe orients women about alarm signs: pain, fever, Vaginal bleeding loss of amniotic fluid and reduction of fetal movement loss of amniotic fluid and reduction of fetal movement.			
17	Observe counsels about nutritional need.			
18	Observe prescribes iron and folic acid.			
19	Observe administered TT injection.			
20	Observe informs mothers about side effects of medicines during pregnancy.			
21	Observe orients women breast feeding, baby vaccination and use of contraception.			
	**Observe on laboratory evaluation**	None	Not done	
1	Hemoglobin test			
2	Grouping and cross matching test			
3	Rhesus factor test			
4	VDRL test			
5	Syphilis test			
6	Hepatitis B test			
7	HIV testing and counseling			
8	Stool examination			
9	Observe on communication with client throughout the procedure and gives her feedback			

	on findings of physical, obstetric and any other procedures done.			
10	Observe Insist her to ask ensure client has understood.			
11	Observe schedules the next appointment according to health needs and women's convenience			
12	Observe records all findings, assessments, diagnosis, and care with client.			
13	Observe thanks clients for her times.			

Annex VI: አማርኛ መጠይቅ

በኮተቤሜትሮፖሊታንዩንቨርሲቲእናበዳግማዊሚኒሊከጤናሳይንስየጤናአገልግሎትአስተዳደርአ ጠባበቅትምህርትክፍልየመላሾችየመረጃቅጽ፤

ጤናይስጥልኝእንደምንነዎት?

የመጣሁትከኮተቤሜትሮፖሊታንዩንቨርሲቲበዳግማዊሚኒሊክጤናሳይንስሜዲካልኮሌጅበማስተ ርስፕሮግራምየጤናአገልግሎትአስተዳደር(Healthservicemanagement)ክፍልተማሪየሆነቸውንገነ ትአጥላባቸውወክዬነው።

በጤናተቋማትየቅድመወሊድክትትልጊዜየሚሰጣቸውየአገልግሎትጥራትየጥናትላይእርስዎበዚህ ጥናትላይእንዲሳተፉተመርጠዋል።የዚህጥናትአላማበአዲስአበባዉስጥየሚገኙጤናጣቢያዎችዉስ ጥየሚሰጠዉንየቅድመወሊድክትትልጥራትለማሻሻልጠቃሚሃሳቦችንለማሳሰብሲሆንከእርስዎየ ምናገኘዉሃሳብትልቅአስተዋጽያለዉመሆኑንእየገለጽንበዚህምርምርውስጥእርስዎሲሳተፉየሚሰ ጡትንሃሳብሚስጥራዊነቱበከፍተኛሁኔታየተጠበቀእንደሆነእያረጋገጥን።በሚሰጡትማንኛውምአ ስተያየትእናየመልስየመረጃወረቀትላይየእርስዎስምበፍፁምአይፃፍም።በስምዎፈንታሚስጥራዊየ መለያቁጥርየሰጥዎታል።ይህእርስዎየሚሰጡትመረጃየሚታወቀውመረጃውንበሚሰበስበውሰው ብቻሆኖመረጃውሚጢርነቱእንደተጠበቀበተዘጋጀውቦታተቆልፎይቀመጣል።መልሱንከአማራጮ ቹየእርስዎንትክክለኛሁኔታየሚገልጸዉንእንዲመልሱልንበትህትናእንጠይቃለን።ተስማምተዋል? የጥናቱንገለፃአዳምጬተረድቼዋለሁለመሳተፍተስማምቻለሁ።

ቃለ-መጠይቅ ያካህደው ሰው ስም--------------------- ፊርማ................ቀን................

አመሰግናሁ!!

አማርኛ መጠይቅ

ክፍል 1: የማህበራዊ ፣ ስነ-ሕዝብና እርግዝና መረጃ መሰብሰቢያ መጠይቆዎች

ተ.ቁ	ጥያቄዎች	መልስና መለያ
101	እድሜ	................አመት
102	ሐይማኖት?	1.ኦርቶዶክስ<br>2. ሙስሊም<br>3.ፕሮቴስታንት<br>4. ካቶሊክ<br>5. ሌላ ይገለፁ ------
103	የጋብቻ ሁኔታ?	1. ያገባ<br>2. ያላገባ<br>3.የፈታ<br>4.የሞተባት<br>5. የተለያዩ
104	የትምህርት ደረጃ	1.ያልተማረ<br>2.አንደኛ ደረጃ ት/ት<br>3.ሁለተኛ ደረጃ<br>4.ዲፕሎማ<br>5.ዲግሪ<br>6. ሁለተኛድግሪ 7. ስስተኛዲግሪ
105	የስራ ሁኔታ?	1.የመንግስትሰራተኛ2. መንግስታዊያልሆነ

		3. የግልተቀጣሪ<br>4. ተማሪ<br>5.የቀንሰራተኛ<br>6. የቤትእመቤት
10<br>6	በአማካኝ የሚያገኙት የወር<br>ገቢዎት ምን ያህል ነው;	------------------------
	ስንት ግዜ አርግዘው ያውቃሉ	------------------
	ያአሁኑእርግዝናዎአቅድዎወይምማርገዝፈል<br>ገዎነው?	1.አዎ<br>2. አይለም
	ከዚህየጤናድርጅትለቅድመወሊድክትትልምን<br>ያህልጊዜመጡ?	1.ለመጀመሪያጊዜ2.ሁለተኛጊ<br>ዜ<br>3.<br>ሶስተኛጊዜ4.ከሶስትጌዜበላይናበ<br>ተደጋጋሚ

ክፍልሁለት፡- ከስነ- ተዋልዶ ጋር የተያየዙ ጥያቄዎች

ጥያቄዎች	መልስና መለያ)
የአሁንእርግዝናዎእድሜውስንትነው?	1. --- ወር3. አላውቅም<br>2.---- ሳምንት
የመጀመሪያቅድመምርመራዎመቼነበር	1. -------ቀን2.ወር----<br>3.አላውቅም
በአሁኑእርግዝናዎትክትትልማድረግለምንወሰኑ?	1.የመንጋጋቆልፍክትባትለማግኘት<br>2.የቅድመወሊድክትትልለማድረግ<br>3.የነፍሰጡርምርምርለማድርግ<br>4.ሌላየጤናምርምርለማድረግ<br>5. ሌላካለግለጥ----------------
የጤናባለሞያው /ዋበዚህክትትልወቅትጠቅላላምርመራአድርገውልታል?	1.አዎ 2. የለም
የተ. ቁ 1013 መልስአዎከሆነከሚከተሉትየትኛውንምርመራአድርገውልታል?	
1.ክብደትተለክትዋል	1.አዎ2.የለም 3. አላውቅም
2.አይንመንጣት(pallor) ታይተዋል	1.አዎ2.የለም

3. የድምግፌትተለክትዋል	1.አዎ2.የለም 3. አላውቅም
4.ዕብጠትእንዳለብዎታይተዋል	1.አዎ2.የለም 3. አላውቅም
5. አልትራሳውንድ	1.አዎ2.የለም 3. አላውቅም
የጤናባለሙያሙሉምርመራበሚያደርግሎትጊዜው ጤቱንነግሮዎታል?	1.አዎተነግሮኛል2.አልተነገኝም3 .አላውቅም
የ1014- መልሶአዎከሆነምርመራከማድረጉበፊትገለፃተነግሮ ታል?	1.አዎተነግሮኛል2.አልተነገኝም
ከዚህበታችየተዘረዘሩትንእንዲሰሩወደለላቦራቶሪም ርመራተልከዋል?	
1. የደምማነስ (Hg/HCT)	1. አዎ 2. የለም
2. ቪ.ዲ.አር.ኤሌ (VDRL)/ቂጥኝ	1. አዎ 2. የለም
3. የደምዓይነት (blood group)/	1. አዎ 2. የለም
4. አር. ኤችፋክተር (RH)/ ሾተላይ	1. አዎ 2. የለም
5. የሽንትምርመራ/	1. አዎ 2. የለም
6. ኤች.አይቪ(HIV) /	1. አዎ 2. የለም
7. የሰገራምርመራ	1. አዎ 2. የለም
8. ሌላካለግለጥ-------	
.አይረን/ፎሌትተሰጥትዋል	1.አዎ 2. የለም 3. አላውቅም
ተ. ቁ1018 መልሶዎአዎለምንይጠቅመውታል	1. የደምማነስንይከላከላል 2. ለጥንካሬ 3.መውሰድአያሰፈልግም 4. አላውቅም
በዚህእርግዝናየመንጋጋቆልፍክትባትተዎግተዋል	1.አዎ2.የለም 3. አላውቅም
ተ. ቁ1020መልሶዎአዎለስንትጊዜወሰዱ	------------- ጊዜ
በምርመራዎወቅትከማንኛዉምአካል / ሰውሚስጥርዎተጠብቋል?	1.አዎ2.የለም 3. እርግጠኛአደለም

በዚህበጉብኝትዎወቅትየአገልግሎትሰጪዉአቀባበ ልትሁትነበር?	1. አዎ 2. አደለም 3. አላውቅም
ለምርመራጤናድርጅቱከደረሱበኋላህኪምዘንድለመ ቅረብምንያህልጊዜፈጅቶብዎታል?	1. ---ደቂቃ 3. ------ቀናት<br>2. ----ሰአት4.ኣላስታውስም
ከላይስለቆዩበትሰዓትምንይሰማሃል/ደስተኛነዎት?	1. በጣምየማያረካ<br>2. የማያረካ<br>3. አላውቅም<br>4. የሚያረካ<br>5. በጣምየሚያረካ
ወደጤናድርጅትለክትትልመጥተዉህክምናሳያገኙየ ተመለሱበትጊዜአለወይ?	1. አዎ<br>2. የለም
የተ. ቁ.102መልሰዎአዎከሆነምክንያቱንይግለጹ	1.ባለሙያሰላነበረ<br>2.የላብራቶሪእቃሰላነበረ<br>3.እረጅምሰአትስለጠበኩ 4. ልሌላካለ-
በቅድመወሊድምርመራወቅትበአብዛኛዉክትትልየ ተደረገለዎትበስንትባለሙያዎች?	1. አንድነበረ<br>2. ሁለትነበሩ<br>3. የተለያዩሰዎችነበሩ-
በነዚህባለሙያዎችበተደረገልዎክትትልምንያህልእ ረክተዋል	1. በጣምየማያረካ<br>2. የማያረካ<br>3. አላውቅም<br>4. የሚያረካ<br>5.በጣምየሚያረካ
በክትትልወቅትምርመራዎንያደረጉልዎትባለሙያ ዎችበአብዛኛዉንጊዜየትኞቹነበሩ	1. አዋላጆች<br>2. ነርስ<br>3. ሀኪሞች.<br>4. ጤናመኮን

	5. ነርስና አዖላጅ<br>6. አላውቅም
ከተጠቀሱትባለሚያዎችበየትኞቹክትትልቢደረግል ዎበጣምይረካሉ/ ደእሰተኛይሆናሉ?	1. አዋላጆች<br>2. ነርስ<br>3. ሀኪሞች<br>4. ጤናመኮን<br>5. ነርስናአዖላጅ
በቅድመወሊድክትትልወቅትይከታተሉዎትየነበሩባ ለሙያዎችምንያህልከዚህበታችየተዘረዘሩትንበበቂ ሁኔታአብራርተዉልዎታል?	1.የላቦራቶሪምርመራ-----<br>2.በእርግዝናጊዜሊከሰቱየምችሉ ዉስብስብችግሮች<br>3. ደምመፍሰስ--------.<br>4. የራስህመምናብዥታ----<br>5.የእውነተኛየመጀመሪየምጥደረ ጃምልክቶች--------<br>6.ለመዉለድመቼወደህክምናተቋ ማትመሄድእንዳለበዎት-<br>7.ምንቅድመዝግጅትማድረግእን ዳለብዎ-
ቅድመወሊድምርመራብተመለከተከሚከተሉትበየት ኞቹይስመማሉ?	1.የሚከታተሉኝንባለሙያዎችለ ማግኘትብዙጊዜመጠበቅአልነበረ ብኝም. ----------<br>2.በየጊዜዉየተለያዩባለሙያዎች ከሚከታተሉኝይልቅበአንድባለ ሙያበቋሚነትቢደረግልኝጥሩይ መስለኛል. -------------<br>3.ባለሙያዎችጥያቄእንድጠይቅ ይገፋፉኛል-

	4.የሚከታተሉኝባለሙያዎችለመ ነጋገርበቂጊዜነበ .----
በክትትልዎትወቅትባለሙያዎችበእርግዝናወቅትሊ ከሰቱስለሚችሉአደገኛምልክቶችወይምአስቸኳይእ ርዳታስለሚስፈልጋቸዉዉስብስብየጤናችግሮችነግ ሮታል?	1. አዎን 2. የለም<br>3. ትዝአይለኝም
የተ.ቁ 1034<br>መልሶአዎከሆነአገልግሎትየሰጥትባለሙያስለየትኛ ውጉዳይበቂ (አመርቂ) መረጃሰጦት?	
1. በእርግዝናጊዜመድማት	1. አዎ<br>2. አልስጠኝም<br>3. ትዝአይለኝም
2.የሽንትውሃመፍሰስ	1. አዎ<br>2. አልስጠኝም<br>3. ትዝአይለኝም
3.ከባድየራስምታት	1. አዎ<br>2. አልስጠኝ<br>3. ትዝአይለኝም
4. የእይታብይታ	1. አዎ<br>2. አልስጠኝ<br>3. ትዝአይለኝም
5.ትኩሳት	1. አዎ<br>2. አልስጠኝ<br>3. ትዝአይለኝም
6.ከባድየሆድቁርጠት/ ህመም	1. አዎ<br>2. አልስጠኝ<br>3. ትዝአይለኝም
7. መንቀጥቀጥ	1. አዎ

	2. አልስጠኝ<br>3. ትዝአይለኝም
በዚህእርግዝናክትትልዎትወቅትባለሙያዎችየቅድ ሚያወሊድዝግጁትት/ርትተነግሮታል	1. አዎ<br>2. አልስጠኝ<br>3. ትዝአይለኝም
የተ.ቁ 1036 መልሶአዎከሆነአገልግሎትየሰጦትባለሙያስለየትኛ ውእቅድ /ጉዳይበቂ (አመርቂ) መረጃሰጦት?	1.የመውለጃ ቦታ---------<br>2. ለወሊድ የሚስፈልጉ እቃዎች-----<br>3. ስለትራንስፖርት<br>4.ለድንገተኛ የሚያስፈልግ ገንዘብ<br>5. እገዛ የሚረግ ሰው ማዘጋጀት-------<br>6. ደም የሚለግስ ማዘጋጅት
በአጠቃላይበዛሬዉእለትበዚህየጤናድርጅትዉስጥያ ገኙትንአገልግሎትእንዴትይገመግሙታል?	1. በጣም የማያረካ<br>2. የማያረካ<br>3. አላውቅም<br>4. የሚያረካ<br>5. በጣም የሚያረካ
ለተሰጦትአገልግሎትምንያህልከፍለዋል	------------- ብር
ክፍያውንእንዴትአገኙት	1.ከአቅምበላይ<br>2.ተመጣጣች<br>3.በጣምትንሽነው
የትመዉለድይፈልጋሉ?	1. ከዚህ ጤና ጣቢያ 3.ቤት ዉስጥ<br>2. በሌላ ጤና ድርጅት<br>4. ሆስፒታል

ምላሽዎ 1 ቁጥርከሆነለምንመረጡት?	1. በአቅራቢያስሆነ<br>2.በባለሙያዎችጥሩግልጋሎትስ ለሚሰጥ<br>3.ሰራተኞቹን ስለምንወደዳቸዉ<br>4.የተሸለየህክምናመሳሪያዎችስላ ሉ<br>5. ሁል ጊዜ ከዚህ የምወልድ ስሆነ<br>6. በአጭር ጊዜ ስለምጨርስ<br>7.ሌላ ካለ------------------
ለክትትልመቼእንደሚመጡተነግሮታል?	1.አዎ<br>2.አልተነገረኝም
ጥሩቅድመወሊድምርመራእንዲኖርመሻሻልአለባቸ ዉየሚሉዋቸዉንይጥቀሱ	1.ባለሙያዎቹንመጨመር<br>2. መድሀኒትአቅርቦት<br>3. የላብራቶሪምርመራ<br>4.የህክምናመስጫዉንቤትንጽህና መጠበቅ<br>5. መቆያጊዜማሳጠር<br>6.ተጨማሪክፍሎችመጨመር
በዛሬዉእለትወይምቀደምሲልየጤናምክርተሰጥተ ዎታልወይ?	1. አዎ<br>2. አልተሰጠኝም
የ1045- መልስአዎከሆነየትኛውን	1.HIV ምርመራ ---------<br>2.አባላዘርበሽታ------------<br>3. የተመጣጠነምግብ -----<br>4.የድህረወሊድክትትል ---<br>5.የቤተሰብምጣኔ --------<br>6. ክትባት ------------

በተሰጠዎትምክርምንያህልእረክተዋል	1. በጣም የማያረካ<br>2. የማያረካ<br>3. አላውቅም<br>4. የሚያረካ<br>5. በጣም የሚያረካ
ከተደረገለዎት ›እንክብካቤዉስጥበጣም ጥሩነዉየሚሉትየትኛዉንነበር	1.የጤናምክር<br>2.የመንጋጋቆልፍክትባት<br>3. HIV ምርመራምክር<br>4.የቤተሰብምጣኔምርመራአገልግ ሎት
በእርግዝናክትትልወቅትያጋጠሞትችግርአለ	1.አዎ<br>2.አላጋጠመኝም
እስከ አሁን በተደረገልዎት አጠቃላይ የቅድመ-ወሊድ ክትትሎች ምን ያህል ረክተዋል	1. በጣም የማያረካ<br>2. የማያረካ<br>3. አላውቅም<br>4.የሚያረካ<br>5. በጣም የሚያረካ
የድህረ- ወሊድ ክትትል ጊዜ አገልግሎት ያኙት እንደሚፈልጉት/አዳሰቡት ነበረ	1.አዎ<br>2.አልነበርም
